# Barriers and facilitators to safer supply pilot program implementation in Canada: a qualitative assessment of service provider perspectives

**DOI:** 10.1186/s12954-025-01177-0

**Published:** 2025-04-28

**Authors:** Frishta Nafeh, Lucas Martignetti, Carol Strike, Gillian Kolla, Matthew Bonn, Caroline Brunelle, Jade Boyd, Elaine Hyshka, Cassidy Morris, Jolene Eeuwes, Heather Hobbs, Elizabeth Haywood, Bernadette Pauly, Dan Werb, Mohammad Karamouzian

**Affiliations:** 1 Centre On Drug Policy Evaluation, MAP Centre for Urban Health Solutions, St. Michael’s Hospital, Toronto, ON Canada; 2 Institute of Health Policy, Management and Evaluation, University of Toronto, Toronto, ON Canada; 3 Dalla Lana School of Public Health, University of Toronto, Toronto, ON Canada; 4 Faculty of Medicine, Memorial University, St. John’s, NL Canada; 5 Canadian AIDS Society, Ottawa, ON Canada; 6 Department of Psychology, University of New Brunswick, Fredericton, NB Canada; 7 Department of Medicine, Division of Social Medicine, University of British Columbia, Vancouver, BC Canada; 8 School of Public Health, University of Alberta, Edmonton, AB Canada; 9 London InterCommunity Health Centre, London, ON Canada; 10 AVI Health and Community Services, Victoria, BC Canada; 11 Island Health Authority, Victoria, BC Canada; 12 Canadian Institute for Substance Use Research, University of Victoria, Victoria, BC Canada; 13 Division of Infectious Diseases and Global Public Health, University of California San Diego School of Medicine, La Jolla, CA USA

**Keywords:** Harm reduction, Drug overdose, Safer supply, Implementation science, Substance-related disorders

## Abstract

**Background:**

In response to the ongoing drug toxicity crisis, driven by fentanyl and its analogues in the unregulated drug supply, Canada has funded several safer supply programs, which provide pharmaceutical-grade medications to reduce the reliance on toxic unregulated drug supply for people who use drugs. In this study, we examined barriers and facilitators that influenced the implementation of integrated safer supply pilot programs (ISSPP) across Canada.

**Methods:**

Between March 2022 and May 2023, we conducted a qualitative study using semi-structured interviews with key informants from ten ISSPP located in three provinces across Canada. Data analysis and interpretation of findings were guided by the Consolidated Framework for Implementation Research (CFIR). Thematic analysis was used to code transcripts and identify themes.

**Results:**

ISSPP varied greatly in the degree of ancillary and wraparound services provided. Additionally, differences existed across the ten programs in terms of eligibility criteria for enrolling clients and the availability of medication options. We found twelve constructs and three sub-constructs across four domains of CFIR that influenced the implementation of ISSPP. Implementation facilitators included low-barrier and client-centered delivery model, ongoing needs assessment through program monitoring and evaluation, integration of wraparound care, partnership with local services to coordinate client care, community buy-in, clinical protocols and standardized practices, and multidisciplinary care teams with motivated staff. Major barriers to ISSPP implementation were a volatile and toxic unregulated drug supply, complicated policy environments, unsustainable funding models, unsupportive regulatory environments, limited medication options, limited physical space, as well as staff shortage.

**Conclusions:**

Despite several internal implementation facilitators, ISSPP faced many external and policy-level implementation barriers. Future safer supply programs should be guided by evidence-based planning and implementation, drawing from successful experiences in harm reduction implementation. Implementation facilitators, in particular, evidence-based practice guidelines along with better monitoring of client outcomes can be leveraged to enhance quality of care, address client needs and preferences, and mitigate unintended harms.

**Supplementary Information:**

The online version contains supplementary material available at 10.1186/s12954-025-01177-0.

## Introduction

With over 40,000 overdose-related deaths in the past eight years, Canada continues to grapple with a drug toxicity crisis primarily driven by an unregulated, toxic, and unpredictable drug supply dominated by fentanyl and its analogues [1]. In response to the drug toxicity crisis, various federal and provincial efforts have been implemented, including the development of guidelines for opioid prescribing, scaling up harm reduction strategies, such as supervised consumption sites, drug-checking services, take-home naloxone programs, and the expansion of treatment options for opioid use disorder (OUD) [2–4]. Among these initiatives, a new harm reduction strategy known as safer supply has been implemented and rapidly expanded during the COVID-19 pandemic across select sites in Canada, following Health Canada’s allocation of short-term funding [2, 5, 6]. Conceptually, safer supply refers to the provision of prescribed pharmaceutical-grade medications as an alternative to unregulated drugs [5, 7]. Unlike opioid agonist treatment (OAT), safer supply is not designated as a treatment for OUD, but rather a harm reduction strategy to prevent overdose deaths [5, 7]. Safer supply operates within the regulatory framework of the Controlled Drugs and Substances Act (CDSA), which governs the legal distribution and prescribing of controlled substances in Canada [8]. Health Canada issued temporary exemptions under the CDSA to facilitate prescribing amid the dual public health emergencies of drug toxicity deaths and COVID-19 [9]. Under CDSA, healthcare professionals can prescribe certain opioids, such as hydromorphone, as part of the safer supply initiative, provided they adhere to national regulations and provincial prescribing guidelines [8, 9].

There are different models of safer supply across Canada with varying clinical practices, target populations, medication types, and regulatory involvement [5, 10, 11]. A detailed description of these models is provided elsewhere [10, 11]. Health Canada’s safer supply programs (hereafter referred to as integrated safer supply pilot programs [ISSPP]) are integrated within existing community services and aim to reduce reliance on the toxic drug supply and related risk of overdose while increasing engagement with primary care [10]. These programs offer a range of opioid medications, such as hydromorphone and fentanyl patches, with some also providing stimulant medications across different harm reduction and primary care settings with varying levels of wraparound ancillary services such as case management, HIV/HCV testing and treatment, and referral to health care and social support services [2, 10, 11]. In the context of ISSPP, wraparound services refer to the comprehensive, integrated supports that address the health and social support needs of individuals receiving prescribed safer supply medications [5]. Many ISSPP also offer long-acting opioids, such as methadone, slow-release oral morphine, and/or buprenorphine/naloxone to assist with opioid withdrawal symptoms [10, 11].

Available evidence to date shows positive health outcomes associated with safer supply programs, including reduced risk of non-fatal overdose [12, 13], fewer emergency department visits and hospitalizations [14], reduction in use of unregulated drugs [13, 15, 16], and decrease in risky drug use behaviours [17, 18]. Some studies have also reported positive social outcomes, such as fewer encounters with the criminal justice system, improved financial stability, and overall improvements in health and well-being [13, 15, 18, 19]. Despite these benefits, ISSPP have limitations. Studies have reported a lack of medication options to meet the high tolerance levels of some clients regularly exposed to high-frequency fentanyl use, low enrollment capacity, and ongoing accessibility issues, namely limited access to take-home and unobserved doses [10, 11, 20]. Furthermore, concerns exist about the potential diversion of prescribed safer supply medications, including diversion to opioid-naïve populations, and increased drug use initiation and overdose among youth [21, 22].

While several studies have described the implementation experiences of different safer supply models [20, 23–27], there is a lack of comprehensive information about providers’ perspectives on implementation barriers and facilitators of ISSPP across different communities [20, 26, 27]. Studies investigating the implementation experiences of ISSPP report findings that are either focused on pre-implementation or early-implementation phases [20, 26, 27]. Results from these studies are confounded by system-level challenges that the health and social service sector faced due to the COVID-19 pandemic and, therefore, do not capture the dynamics and diversity of implementation experiences after programs were operational. Our study addresses these gaps by systematically examining the factors that influenced the implementation of ISSPP based on the perspectives of service providers, who have thorough first-hand knowledge and experience about their implementation and clinical operations.

## Materials and methods

### Conceptual framework

We used the updated Consolidated Framework for Implementation Research (CFIR) as our guiding framework. CFIR consists of 48 constructs and 19 sub-constructs, grouped into five major domains: innovation (e.g., ISSPP), outer setting (e.g., policies and laws), inner setting (e.g., structural characteristics, culture, available resources), individuals (e.g., characteristics of program clients and staff), and implementation process (e.g., planning, assessing context) [28].

### Study aim, design and setting

This study was part of a larger national evaluation of a select group of ISSPP implemented in three provinces, funded by Health Canada’s Substance Use and Addictions Program (SUAP) [29]. We use the term implementation to capture both factors influencing initial program setup  as well as ongoing operational challenges to acknowledge the dynamic nature of ISSPP and context-specific factors that require ongoing tailoring of implementation strategies [28]. A detailed description of ISSPP is provided in our previous publication [10]. For this study, we used a qualitative descriptive research design [30] with semi-structured interviews with key informants from ISSPP to explore barriers and facilitators to program implementation.

### Participant recruitment and data collection

Eleven ISSPP were selected by SUAP to participate in an evaluation of their services. One program, implemented in an Indigenous community, was evaluated separately to address specific community needs and concerns; the results of this program's evaluation are not discussed in this paper. We used purposive sampling to recruit staff in leadership roles from ten programs. These key informants had been involved in the entire implementation process and possessed detailed knowledge of implementation factors. All ten organizations that were contacted agreed to participate. Participants completed a survey form gathering descriptive information about the ISSPP, including program setting, medications provided, percentage of clients reporting a sufficient dose, medications not currently available that would be helpful, and staff composition. The interview guide was developed through a collaborative effort by an interdisciplinary team of researchers and community scholars with expertise in qualitative methods and implementation science. Informed by the CFIR framework, the interview guide assessed how contextual factors—both internal and external—impacted the implementation and scale-up efforts. It included questions about program goals, client targets, interest-holder relationships, staff capacity, client care preferences, and the impact of COVID-19 on service delivery. After obtaining informed consent, the co-principal investigators (DW & MK) conducted one-on-one interviews in English via Zoom from March 2022 to May 2023. Interviews averaged 75 minutes, were audio-recorded on secure hospital laptops, and were transcribed verbatim by an approved external agency.

### Data analysis

**Fig. 1:**
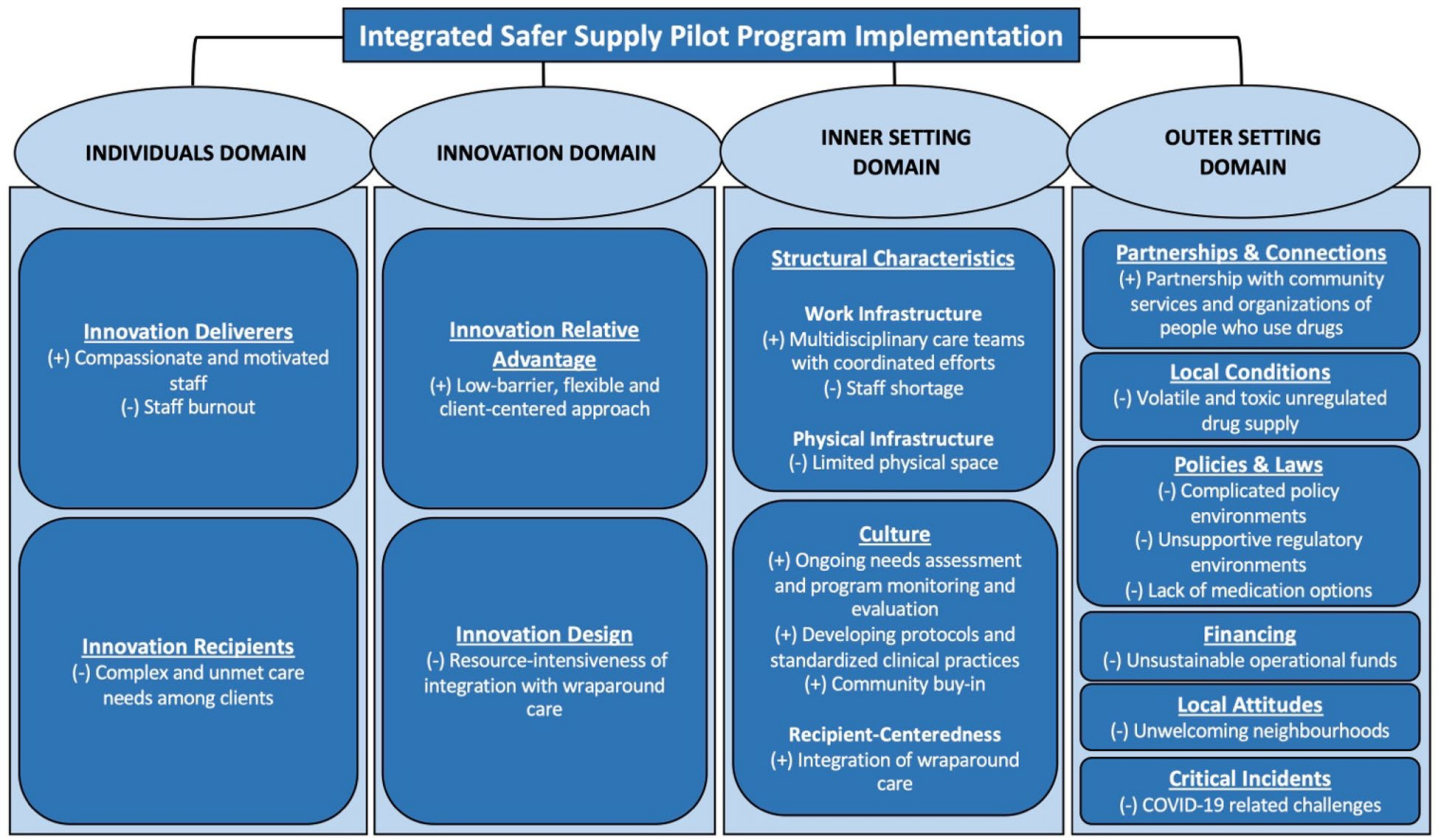
Overview of Identified Themes Informed by the Consolidated Framework for Implementation Research (CFIR). Framework modified from Damschroder et al., 2022 and CFIR website (https://cfirguide.org/constructs/). ( +) indicates facilitator to safer supply pilot program implementation and (-) indicates barrier. CFIR constructs are underlined and bolded. Themes representing barriers and facilitators to safer supply pilot program implementation are listed under relevant CFIR constructs and sub-constructs (bolded)

We summarized and presented descriptive information on the program characteristics gathered through the survey form and semi-structured interviews. We analyzed the transcripts using Braun and Clarke’s six-step thematic analysis guide [31]. Initially, a trained member of the research team (*FN*) read all the transcripts to become familiar with the data. FN then developed initial codes using an inductive approach. Codes were used to develop themes in an iterative process involving discussions with co-authors. A deductive approach was used to organize themes pertaining to barriers and facilitators to ISSPP implementation, guided by relevant CFIR domains and constructs (Fig. 1). To ensure inter-coder reliability, a second coder (*LM*) independently coded 20% of the transcripts. The coders met regularly until coding disagreements were resolved and a consensus was reached. NVivo version 14 was used for data analysis. The findings were shared with key informants for member-checking and validation of results [32]. The consolidated criteria for reporting qualitative research (COREQ) checklist was used to inform our methodological reporting [33]. See additional file 1 for the COREQ checklist and additional detail about our study methods.

### Ethics statement

Ethics approval for this study was obtained from the Unity Health Toronto Research Ethics Board (Project #21–273).

## Results

### Description of ISSPP

Table 1 presents a descriptive overview of ISSPP included in this study. These programs were implemented across various settings, including standalone clinics, primary care clinics, harm reduction and supervised consumption sites, and through biometric vending machines. Program eligibility criteria for clients typically included individuals who were dependent on unregulated fentanyl or opioids and who were deemed to be at high risk of overdose. Some programs also required participants to report engaging in daily fentanyl use, while others limited participation to individuals with previous experience in OAT.

**Table 1:** Descriptive summary of integrated safer supply pilot programs (*n* = 10)

Organization	Program setting (integrated/stand-alone)	Medication provided	Percentage of clients reporting sufficient dose	Medications not currently available that would be helpful	Program staff	Eligibility criteria	Program goals and outcomes
	Other services provided						
#1	**Program setting**	**Opioids**	Some (25–50%)	Injectable hydromorphone, diacetylmorphine & fentanyl formulation	Physician Prescriber (*n* = 6PT); Clinical Nurse Lead (*n* = 1FT); Registered Nurse (*n* = 3FT); System Navigator (*n* = 2FT); PWLLE Support Worker (*n* = 4PT); Project Director (*n* = 1FT); Medical Office Administrator (*n* = 1FT)	Dependent on street/unregulated fentanyl and at high risk of drug poisoning; have not been able to reduce overdose risk via OAT or risk mitigation guidance; high tolerance to opioids; not already connected to a physician or nurse practitioner	Reduce fatal and non-fatal overdoses
	Stand-alone safer opioid supply clinic	*Non-observed:*		Smokeable diacetylmorphine & fentanyl formulation			Connect people who are not being reached or retained by traditional substance use services and treatment programs
	**Other services provided**	Hydromorphone short acting (tablet); Hydromorphone controlled release (capsule); Oxycodone (tablet); Morphine sulfate (capsule, 12-h formulation)		Cocaine & methamphetamine			Generate evidence for safer supply
	Onsite primary care & referral; HIV/HCV testing; harm reduction equipment distribution; overdose prevention site/SCS; onsite & referral to social services; referral to specialized healthcare; individual/group support	*Observed:*					
		Morphine sulfate (capsule, 24-h formulation); Fentanyl (tablet); Sufentanil (injectable); Methadone (liquid); Buprenorphine (tablet)					
		**Psychostimulants**					
		*Non-observed:*					
		Dexedrine (tablet); Methylphenidate (tablet)					
#2	**Program setting**	**Opioids**	Some (25–50%)	Injectable hydromorphone, diacetylmorphine & fentanyl formulation	Physician Prescriber (*n* = 1FT); Nurse Prescriber (*n* = 1FT, n = 1PT); Registered Nurse (*n* = 5FT); Social Worker (*n* = 1FT); Outreach Worker (*n* = 4FT); Patient Navigator (*n* = 1FT); Care Facilitator (*n* = 2FT); Clinic Manager (*n* = 1FT); Program Coordinator (*n* = 1PT); Clinic Assistant (*n* = 3FT); In-Reach Worker (*n* = 1FT); Infectious Diseases Care Program Team for HIV/HCV (*n* = 7FT); Lab Technician (*n* = 1FT); Community Paramedics (*n* = 2FT)	Dependent on street/unregulated fentanyl and at high risk of drug poisoning; previously tried multiple other forms of addiction treatment (abstinent based & pharmacological approaches); precariously housed; street-involved in terms of sex work; involved in criminal activity; has infectious diseases (uncontrolled HIV & HCV)	Pathway to care model to achieve medical and social stability
	Safer opioid supply embedded within an existing primary care clinic	*Non-observed:*		Smokeable diacetylmorphine			Engage clients with primary care through a community health centre model
	**Other services provided**	Hydromorphone short acting (tablet); Fentanyl patch; Hydromorphone (injectable); Buprenorphine (tablet)		Stimulant options			
	Onsite primary care; onsite HIV/HCV testing & treatment; harm reduction equipment distribution; onsite & referral to social services; referral to specialized healthcare; case management; individual/group support; cultural programming	*Observed:*					
		Methadone (liquid)					
		Both observed & non-observed:					
		Hydromorphone controlled release (capsule); Morphine sulfate (capsule, 12- & 24-h formulations)					
		**Psychostimulants**					
		*Non-observed:*					
		Methylphenidate (tablet)					
		**Benzodiazepines**					
		*Non-observed:*					
		Diazepam (tablet); Clonazepam (tablet)					
#3	**Program setting**	**Opioids**	All/almost all (76–100%)	Smokeable diacetylmorphine	Physician Prescriber (*n* = 1PT); Nurse Prescriber (*n* = 1PT); Registered Nurse (*n* = 1PT); Outreach Worker (*n* = 4FT); Patient Navigator (*n* = 4FT); Program Coordinator (*n* = 1FT)	Current fentanyl use (daily)	Reduce reliance on street drugs to ultimately prevent overdose deaths
	Safer opioid supply biometric machine located in a housing unit and an existing overdose prevention site	*Non-observed:*					Reduce criminal activity engagement and chaos in clients’ lives (achieve socioeconomic stabilization)
	**Other services provided**	Only providing Hydromorphone short acting (tablet), dispensed through biometric machine					Reduce overall drug use in the long-term
	Referral to primary health care and social services						
#4	**Program setting**	**Opioids**	Few (less than 25%)	Injectable hydromorphone, diacetylmorphine & fentanyl formulation	Nurse Prescriber (*n* = 2FT); Registered Nurse (*n* = 4FT); Case Manager (*n* = 2FT); Clinic Manager (*n* = 1FT); Project Manager (*n* = 1FT); Staff Supervisor (*n* = 1FT)	Daily use of street/unregulated opioids (minimum 5 days per week)	Reduce overdose risk
	Safer opioid supply embedded within an existing primary care clinic	*Non-observed:*		Smokeable diacetylmorphine			Reduce use of street/unregulated drugs
	**Other services provided**	Hydromorphone short acting (tablet); Morphine sulfate (capsule, 24-h formulation)		Higher potency medications			Increase stabilization for clients (anything related to social determinants of health)
	Onsite & referral to primary care; onsite HIV/HCV testing & treatment; harm reduction equipment distribution; overdose prevention site/SCS; onsite & referral to social services; referral to specialized healthcare; case management; individual/group support; cultural programming	*Observed:*					Provide greater connection to primary care and social services
		Methadone (liquid)					Provide linkage to long-term, ongoing care
#5	**Program setting**	**Opioids**	Some (25–50%)	Smokeable diacetylmorphine	Nurse Prescriber (*n* = 1FT); Case Manager (*n* = 1FT); Registered Nurse (*n* = 1FT); Patient Navigator (*n* = 1FT, 2 PT); Office Administrator (*n* = 1FT); Program Manager (*n* = 1FT)	Diagnosis of opioid use disorder and at high risk of drug toxicity death	Reduce drug poisoning death
	Safer opioid supply embedded within an existing primary care clinic	*Non-observed:*		Higher potency medications			Provide access to harm reduction care and harm reduction education
	**Other services provided**	Hydromorphone short acting (tablet); Oxycodone (tablet); Morphine sulfate (capsule, 24-h formulation); Methadone (liquid); Fentanyl patch					
	Onsite primary care; onsite HIV/HCV testing & treatment; harm reduction equipment distribution; onsite & referral to social services; referral to specialized healthcare; case management; individual/group support						
#6	**Program setting**	**Opioids**	Most (51–75%)	Injectable fentanyl formulation	Physician Prescriber (*n* = 1PT); Registered Nurse/Clinic Manager (*n* = 1FT); Licensed Practical Nurse (*n* = 6FT, *n* = 2PT); Case Manager/Social Worker (*n* = 2FT); Outreach Worker (*n* = 1FT, 1PT); Onsite Pharmacist (*n* = 1PT)	Intravenous opioid use; using for more than 6 months; have not been successful on oral replacement therapy	Enhance engagement with community services
	Stand-alone dedicated iOAT clinic	*Observed:*		Injectable hydromorphone in formulations of 100mg/ml			Provide access to primary care, screening and treatment for HCV
	**Other services provided**	Hydromorphone short acting (tablet); Hydromorphone controlled release (capsule); Morphine sulfate (capsule, 12- & 24-h formulations); Hydromorphone (injectable); Methadone (liquid); Buprenorphine-Naloxone (tablet); Buprenorphine extended release (injectable)					Overdose prevention
	Referral to primary care; onsite HIV/HCV testing & treatment; harm reduction equipment distribution; onsite & referral to social services; case management; individual/group support	**Psychostimulants**					
		*Observed:*					
		Dextroamphetamine (tablet); Methylphenidate (tablet); Aripiprazole (tablet); Sertraline (tablet)					
#7	**Program setting**	**Opioids**	Most (51–75%)	Smokeable diacetylmorphine	Physician Prescriber (*n* = 6FT, 12PT); Licensed Practical Nurse (*n* = 4FT, 8PT); Social Worker (*n* = 6FT, 6PT); Case Manager (*n* = 2FT, 2PT); Outreach Worker (*n* = 2FT, 2PT); Patient Navigator (*n* = 6FT, 6PT); Onsite Dietician (*n* = 1FT, 2PT); Clinic Manager (*n* = 2FT, 2PT); Program Coordinator (*n* = 2FT, 2PT); Clinic Assistant (*n* = 2FT, 2PT); Onsite Pharmacist (*n* = 2FT, 2PT)	Only criteria is if they are unattached to care	Reduce street/unregulated drug use
	Safer opioid supply and iOAT embedded within an existing primary care clinic and safe consumption site	*Non-observed:*		Injectable fentanyl formulation			Provide linkage to primary care
	**Other services provided**	Hydromorphone short acting (tablet); Oxycodone (tablet)		Intranasal (inhale) diacetylmorphine			
	Onsite primary care; HIV/HCV treatment; harm reduction equipment distribution; overdose prevention site/SCS; onsite social services	*Observed:*					
		Sufentanil (injectable); Buprenorphine (tablet)					
		*Both observed & non-observed:*					
		Morphine sulfate (capsule, 12- & 24-h formulations); Fentanyl patch; Hydromorphone (injectable); Diacetylmorphine (injectable); Methadone (liquid)					
		**Psychostimulants**					
		*Non-observed:*					
		Dexedrine (tablet); Methylphenidate (tablet)					
		**Benzodiazepines**					
		*Observed:*					
		Diazepam (tablet); Clonazepam (tablet); Alprazolam (tablet)					
#8	**Program setting**	**Opioids**	Some (25–50%)	Injectable diacetylmorphine	Physician Prescriber (*n* = 2PT); Registered Nurse (*n* = 1FT); Licensed Practical Nurse (*n* = 3FT, 2PT); Harm Reduction/Mental Health Worker (*n* = 1FT, 2PT); Clinic Manager (*n* = 1PT); Program Coordinator (*n* = 1FT); Indigenous Community Support Worker (*n* = 1FT); Contingency Management Worker (*n* = 2PT)	Severe opioid use and at risk of overdose death confirmed via positive screen for fentanyl use	Reduce overdose death
	Stand-alone dedicated safer opioid supply program	*Observed:*		Smokeable diacetylmorphine			Reduce use of street/unregulated drugs
	**Other services**	Morphine sulfate (capsule, 12- & 24-h formulations); Fentanyl patch; Methadone (liquid); Fentanyl powder		Smokeable fentanyl formulation (have this product but no designated space for clients to consume)			Provide wraparound care
	Onsite & referral to primary care; onsite HIV/HCV testing & treatment; onsite & referral to social services; referral to specialized healthcare; case management; individual/group support	**Psychostimulants**					
		*Non-observed:*					
		Dextroamphetamine (tablet)					
#9	**Program setting**	**Opioids**	Most (51–75%)	Smokeable diacetylmorphine	Registered Nurse (*n* = 1PT); Licensed Practical Nurse (*n* = 1FT, 1PT); Harm Reduction/Mental Health Worker (*n* = 2PT)	Addiction to opioid injection drug use	Reduce overdose and eliminate cause of death
	Stand-alone TiOAT clinic	*Observed:*					Achieve socioeconomic stability among clients
	**Other services provided**	Hydromorphone short acting (tablet); Fentanyl patch					Connect clients to the care they need
	Onsite primary care; harm reduction equipment distribution; overdose prevention site/SCS; referral to social services; referral to specialized healthcare						
#10	**Program setting**	**Opioids**	Most (51–75%)	Injectable diacetylmorphine & injectable fentanyl formulation	Physician Prescriber (*n* = 1PT); Nurse Prescriber/Supervisor (*n* = 1FT); Registered Nurse (*n* = 4FT); Case Manager (*n* = 2FT); Clinic Manager (*n* = 1PT); Peer Support Worker (*n* = 1FT)	Using opioids in some way and willing to attend for daily visits	Reduce reliance on street/unregulated drugs
	iOAT embedded within an existing harm reduction program	*Observed:*		Smokeable diacetylmorphine			Reduce overdose risk
	**Other services provided**	Morphine sulfate (capsule, 24-h formulation); Fentanyl patch; Hydromorphone (injectable); Sufentanil (injectable); Methadone (liquid)					Provide access to all kinds of other services (wraparound case management support, housing & mental health)
	Referral to primary care; onsite HIV/HCV testing & treatment; harm reduction equipment distribution; overdose prevention site/SCS; onsite & referral to social services; case management; individual/group support	**Benzodiazepines**					Increase connection with service system
		*Non-observed:*					Less reliance on safer supply in the long-term
		Clonazepam (tablet)					

OAT, opioid agonist therapy; iOAT, injectable opioid agonist therapy; TiOAT, tablet injectable opioid agonist therapy; SCS, safe consumption site; FT, full-time; PT, part-time; PWLLE, people with lived/living experience

As shown in Table 1, there were 13 opioid formulations that programs reported prescribing, with several of them also providing prescription stimulants and benzodiazepines. In addition to prescribers (i.e., physicians and nurse practitioners), all programs included in this study reported employing registered nurses, as well as support staff such as social workers, case managers, outreach workers, harm reduction workers, Indigenous community support workers, and peer support workers. Programs reported multiple goals and outcomes for clients, such as reducing the incidence of fatal and non-fatal overdoses, decreasing reliance on unregulated opioids, increasing clients’ connections to healthcare and community services, and improving the overall stability of clients’ lives.

Key informants in this study were program directors, clinic managers, and prescribers. To uphold their privacy, we have not included demographic or site-specific details beyond these broad role categories.

### Themes

Thematic analysis revealed twenty themes across four CFIR domains and twelve relevant constructs and three sub-constructs; of the twenty themes that emerged, eight represented facilitators and twelve represented barriers. Figure 1 presents the barrier and facilitator themes under relevant CFIR domains and constructs based on their definitions [28]. Below, we describe the implementation barrier and facilitator themes in the context of the four CFIR domains.

## Individuals domain

### Facilitators

#### Compassionate and motivated staff

Several key informants described having motivated staff who had compassion, empathy, and a strong commitment to supporting people who use drugs, as illustrated below:

*“The biggest strength of the team is compassion and empathy. One of our nurses, I watched him Wednesday night—we’d been closed for an hour—on the street with a gentleman who’s wheelchair-bound. He worked with him for two hours. We know these people in a deep and intimate way, because we’re able to build rapport, credibility, and trust with the majority of those people.”*

These staff members were perceived as crucial in fostering an environment recognized as inclusive and trustworthy by clients, making programs welcoming and reliable.

### Barriers

#### Complex unmet care needs among clients

Key informants reported that ISSPP clients often required extensive healthcare and social support needs. Providing safer supply medications alongside necessary psychosocial services required significantly more staff and resources than initially anticipated. As a result, some organizations had to adjust their enrollment targets to ensure they could deliver comprehensive care. For instance, one organization lowered its target from 200 to approximately 23 clients due to the substantial staff demand needed to operate the program and provide additional psychosocial services:

*“Even with our 23 folks coming in, no, it’s not sufficient. If we were able to get more than 23 people on our program, we would need more staff. It’s just not sustainable at our staffing level right now. So, if we were at [the initial target of] 200, it would be so overwhelming with our current model.”*

When describing client circumstances that made it challenging to reach and engage them in programs, one key informant emphasized that housing insecurity and other structural barriers made it difficult to reach, engage and consistently support some clients:

*“Many of our participants are either homeless or inadequately housed, and that’s just added another layer of complication around connecting with those folks and making sure that they have the resources that they need, and physically finding them to give them their meds.”*

Key informants reported hiring people with lived/living experiences of drug use in outreach roles to enhance program reach and client engagement.

#### Staff burnout

Key informants reported that staff burnout and work-related post-traumatic stress, stemming from grief, loss, and overdose impacts, were common challenges affecting staff retention in programs:

*“We have staff who have absolutely been affected by loss. That’s contributed to folks having to take a break or step back or get some support. We have had some staff with pre-existing PTSD from the overdose crisis, from working through that, and have had to leave.”*

Key informants described various staff support strategies, including peer support, acknowledging work-related stress, offering mental health breaks, and providing skill-enhancing educational webinars.

## Innovation domain

### Facilitators

#### Low-barrier, flexible, and client-centered approach

Key informants emphasized ISSPP’s advantages over traditional addiction programs, including flexibility, low access barriers, and client-centered approaches. They highlighted a culture valuing client self-determination and inclusion of people with lived/living experience in staffing and advisory roles.

*“We’re looking to change how the observed program is run. We consulted with [client] advisory especially with folks who are on observed arm [doses] to see what the pros and cons of change would be, to integrate their feedback into program design. So much goes to advisory, titration, missed doses, any protocols around that.”*

Compared to traditional addiction medicine, ISSPP were described as less punitive and restrictive. They featured more relaxed policies on urine drug screening and did not penalize clients for missed doses:

*“If people are doing well, they’re picking up their pills every day, we don’t hassle them. We want a urine sample when they can give it every two or three months, but it’s not punitive. People want to give it most of the time to show that they’re not using [unregulated] fentanyl anymore. I think the model we’ve established is adequate, that people feel supported when they need it, but we don’t go out of our way to kind of track people down and make them change.”*

Key informants noted that the flexible, client-centered model allowed programs to adapt and improve, addressing clients' evolving needs amid a volatile unregulated drug market.

### Barriers

#### Resource-intensiveness of integration with wraparound care

Key informants stressed the importance of integrating wraparound care, encompassing healthcare and social support services. The provision of these ancillary services varied across sites, with some programs offering them in-house and others referring clients to external organizations. Offering wraparound care increased resource demands on programs in terms of labour and physical space, leading to an increased workload for staff due to clients’ multiple unmet healthcare needs:

*“I think sometimes that’s hard for both staff and participants, because we’re working with folks who are so vulnerable and have so many basic needs that are not being met. We don’t have the staff to sufficiently support people in all of that but do our best.”*

To address these challenges, key informants described leveraging referrals and coordinating care with other community healthcare organizations. Additionally, some reported changing their enrollment criteria, shifting from focusing on individuals at extremely high risk of overdose to a less restrictive criteria to avoid a large influx of clients with complex care needs.

## Inner setting domain

### Facilitators

#### Integration of wraparound care

Key informants reported integrating wraparound care as a critical component of safer supply provision, connecting clients to primary and secondary health services, including HIV/HCV testing and treatment, wound care, and referral to housing support services. Some key informants believed this played a vital role in making programs more appealing to policymakers:

*“This is why I said we integrated into a primary care service. So, the Ministry [Ministry of Health] will have a hard time saying ‘no, we cannot fund this.’ But leaving, say, 4000 people unattached to care? That’s something that I think politicians are not going to like to do.”*

Key informants believed integration of wraparound care enabled ISSPP to serve as a pathway to essential health and social services for their clients, while also making programs more acceptable to those who opposed the idea of safer supply.

#### Multidisciplinary care teams with coordinated efforts

The inner setting’s work infrastructure was crucial for successful ISSPP implementation and operation. Key informants emphasized the importance of multidisciplinary staff teams capable of addressing clients’ diverse psychosocial needs. One informant provided a detailed description of their team’s coordinated approach to care provision:

*“We have a very strong psychosocial team that is composed of a social worker, a peer navigator or a peer support worker, and a behavioural psychologist who do the initial assessment to ensure access to housing and income supports to really address the most pressing needs of the clients. So, it’s a functional team rather than everybody doing their little thing; this is very integrated and there’s a lot of communication happening and coordination.”*

Key informants deemed multidisciplinary teams crucial for offering a wide range of services as part of an integrated safer supply model.

#### Ongoing needs assessment and program monitoring and evaluation

Key informants identified cultivating a learning-centered organizational culture as crucial for running a successful program. This approach, characterized by continuous monitoring and evaluation, enabled the identification of care provision gaps and facilitated program adaptation to align with changing client preferences and clinical advancements:

*“We’ve done some of that program evaluation work with a couple of different researchers and evaluators just to stay on top of our quality and improvement. We have an ongoing developmental evaluation that provides us feedback every six to nine months for program improvement. It’s two steps forward and one step back; we have to assess the environment again and consult with clients.”*

Key informants’ descriptions of the implementation process revealed that ISSPP were dynamic and in a constant state of learning and adapting to be responsive to the needs of clients amid an evolving unregulated drug market:

*“Before we opened, we had a consulting company go to all the local shelters, and we asked future clients what they would like to see. So, the consulting company went out and asked for their feedback … right down to what hours, what ‘kind of drugs would you like to see’. So, the majority of [organization name] is based on what participants originally wanted, and now we’re just adapting things, based on how this stuff changes.”*

Several key informants emphasized the benefits of this data-informed decision-making approach, noting that it ensured programs remained relevant and acceptable to clients.

#### Developing protocols and standardized clinical practices

Key informants reported developing clinical protocols and standardized practices to ensure consistent service delivery. One key informant believed that standardized clinical protocols would be attractive to potential funders:

“*I think because we have a well-refined program model with explicit protocols and a very clear practice, it’s a very marketable framework for continued funding. One of the beautiful things that has been accomplished in the last few months is a fulsome set of operational and clinical protocols, so that everybody can be trained on the same thing. And there’s consistency with service delivery.”*

#### Community buy-in through knowledge exchange with external interest-holders

Several key informants reported engaging with external interest-holders, including local authorities and community organizations, to facilitate knowledge exchange and gain community buy-in for safer supply dialogue:

*“We do a lot of different site visits with all different leadership members of different organizations, but really focusing more on the school board, and the mayor, and different council people, and bylaw, and the police. Because I think that when you’re trying to enlighten people about what the program is. It would be a lot easier to kind of navigate through that if we had someone from the College of Physicians or Nursing.”*

### Barriers

#### Staff shortage

Key informants reported challenges in recruiting physician prescribers and nurse practitioners. Additionally, several key informants stated that recruitment and retention of nursing staff was a significant challenge, as illustrated by the following organization:

*“I think we’re facing nursing shortages across the board. I think it’s been tough to retain and hire new staff. Yeah, we’ve had a number of nursing staff go on leave or leave.”*

The ability to increase staff coverage was mainly dependent on funding, which key informants noted they had limited access to. Some key informants mentioned relying on referral networks to connect clients with community health services when discussing staff coverage issues.

#### Limited physical space

Several key informants from integrated harm reduction programs highlighted inadequate physical space as a significant barrier to delivering comprehensive care, particularly with client intake, observation, and group support within the integrated safer supply model. Inadequate physical space led to crowding inside the clinics, especially for programs providing observed doses:

*“Our space is so small that it gets congested so easily. We don’t have any offices for our case managers to meet with somebody one-on-one. There’s one medical treatment room that I typically see people for wound care or hepatitis C treatment and that room is also used for one-to-one counselling. So, if there’s more than two clients that needs to be met one-on-one, then our clinic is full.”*

The key informant from this organization reported meeting clients in a nearby parking lot for wellness check-ins when the clinic was full, leading to criticism and community pushback.

## Outer setting domain

### Facilitators

#### Partnership with community services and organizations of people who use drugs

Partnerships with local harm reduction agencies, organizations of people who use drugs, and community health services were crucial for program implementation. These partners advised on program design and shared care provision via referrals and collaborations:

*“We’ve got a number of partners – other service providers – that we work with to support mutual clients. We have a pretty direct relationship with a team that provides support to pregnant and parenting folks who use substances and have developed a direct referral process to be able to support folks that they’re working with, who they think could be better supported with the kind of safe supply options that we can offer.”*

### Barriers

#### Volatile and toxic unregulated drug supply

Key informants reported that the dynamic, unpredictable nature of the unregulated drug market posed significant challenges to program goals and meeting client needs, given the market’s evolving and volatile nature with varying potencies of fentanyl and its analogues:

*“The toxicity of the drugs on the street and the complexity of the poison in them is so bad that we need alternatives, because every time we up the dosage, the dose on the street… It’s not because we’re upping it. It’s because the different producers are competing. We’ve hit that ceiling with some of our most challenged clients over and over.”*

Key informants reported an increase in benzodiazepine adulterants in the unregulated fentanyl supply, expressing significant concerns about the prevalence of complex overdoses. This has led to an increased demand for space and staff support in programs integrated into overdose prevention/safe consumption sites:

*“The major one that I’m sure you’ve heard is the benzos in the fentanyl. So we’ll give someone Naloxone and they won’t come out of it, and you know, we’ll have to monitor people for half an hour…and so the increase in calls to 911 to come and get people because we can’t just monitor them.”*

In response to these challenges, key informants reported that programs increased doses to attempt to match clients’ tolerance levels.

#### Complicated policy environments

Key informants reported positive relationships with local community health and social service providers, but inconsistent support from different levels of government. This included mayors, city councils, provincial ministries of health, federal government programs, local health authorities, and local police. Support for ISSPP initiatives often depended on individual opinions or small groups, leading to fluctuating support levels due to changes in leadership or shifting political ideologies about harm reduction:

*“It depends on what constellation of councillors we have and the balance. It’s always about a balance and who tips the balance on Council. In the past, it’s been very supportive—well, it’s been supportive enough that we were able to get supervised injection service through Council in 2017. If we were to go forward today, I don’t know.”*

A common belief among key informants was that there was a lack of collective support for ISSPP from policymakers.

#### Unsustainable operational funds

Key informants reported that limited and unsustainable funding impeded comprehensive, integrated care provision, impacting various programs’ abilities to expand space and hire additional staff. Furthermore, some key informants highlighted the short-term nature of the funding obtained, with no assurance of future extension:

*“We have time-limited funding, and we don’t have any indication about whether or not that’s going to be extended or whether [Provincial Health agency] would pick up the whole program. So, I would say no, in that we just don’t have certainty. We have a temporary period.”*

Another key informant criticized the short notice they received regarding funding renewal and its implications for staff recruitment and retention as well as other program issues:

*“We only learned that we were going to receive another year of funding in February, and our funding ended at the end of March. So, if you’re a staff person there, you’re not staying, you know, if you can find something else. So having short-term funding arrangements really interfere with retention of staff.”*

Additionally, some key informants noted that the politicized nature of safer supply initiatives raised doubts about the long-term viability of these programs, particularly in the context of forthcoming elections:

*“A change in politics—or not a change in politics. I’m thinking about the June election provincially. I think of the time remaining for our funded period without, you know, federal extension, and I think about the hiring landscape and the retention landscape. Like, right now if anyone resigns, we can’t commit to anything beyond March 31, 2023, and nobody wants a ten-month contract.”*

Most key informants identified uncertainty about future funding as one of the greatest threats to program sustainability. Most key informants cited SUAP as the sole source of financial support and saw little to no hope of obtaining funding from their provincial government or local or regional health authorities.

#### Unsupportive regulatory environments

When asked about the influence of provincial regulators on safer supply programming, most key informants focused on the potential influence of colleges of physicians. Some acknowledged their provincial college of physicians’ supportive statement but believed it lacked genuine support behind the scenes. This perception was informed by key informants’ observation of their prescribers’ apprehensions about prescribing higher doses or certain types of opioid medications:

*“Our physicians are always a little worried that they’re out on a limb, especially in certain situations where they might be really trying to address someone who has got high need. Looking at the dose that will make them comfortable becomes a bit of a balance of, ‘am I going to be too far out there? Will I get audited?’ and wanting to make sure that the client feels comfortable knowing clinically that this client might be needing a higher dose. But because of this other pressure, I think it does result sometimes in people not getting the dose that they need.”*

Another key informant reported that their regulatory college recommended ceasing oxycodone prescribing–a situation they described as “*really distressing*” for both clients and the program:

*“Our physician was told—in this process of the audit that’s happening right now—by somebody from the College that he should stop prescribing oxycodone. And this affects at least half of our participants, [who] are on some sort of oxy script. This is really distressing. It affects, as I say, almost half of our participants.”*

A few key informants from select provinces believed that some physicians felt constantly monitored by their regulatory college and feared repercussions, such as losing their medical license or prescribing privileges, following an audit. They described the audit processes as demanding and time-intensive for physicians:

*“They [physician prescribers] are also concerned that this could potentially turn into endless audits and incredible amounts of time. Our doc who is going through his audit right now estimates, at least 12 to 16 hours of administrative work so far just to complete his audit. So, they’re just worried about things like at least part of their license being revoked, and also just being mired in constant surveillance and auditing.”*

Key informants who discussed audits believed that safer supply prescriber audits were due to their college’s concerns about the diversion of prescription opioids:

*“I have definitely come into a lot of fear around diversion, which I found a little confusing at first. There seemed to be a lot of real fear around it.”*

Those who discussed diversion did not perceive it as a serious issue as they reported believing that prescribed safer supply medications were diverted to other individuals who needed them and that concerns about diversion were therefore based on “*nothing*” but “*just fear*”:

*“I am not concerned about diversion. People are very afraid of that [referring to diversion]. I don’t know what’s informing that, though, because it’s not based on anything other than just fear. The drugs are actually too good to end up just sort of kicked, like, to whoever. If it does get diverted, my understanding is that it’s going to get diverted to a fentanyl user.”*

Among key informants who reported their physician(s) undergoing an audit, none reported audits resulting in findings of noncompliance or concerns regarding the physicians’ approaches to prescribing safer supply.

#### Unwelcoming neighbourhoods

Negative perceptions of harm reduction were identified by some key informants as a barrier to implementing programs. Key informants noted instances where some organizations in the neighbourhood voiced hostility regarding the visibility of unhoused people and people who use drugs in their neighbourhoods:

*“They [Business Improvement Area] hate us. They hate us! They wish we were not here. They have such a distorted idea of what our job actually is, so yeah, there’s a lot of negative conversation back and forth. They also really don’t want us on their property, so they don’t want the staff on the property any more than they want folks on there.”*

Unwelcoming neighbourhoods posed challenges for implementing and operating ISSPP:

*“We are planted right in a busy business area. So when people see the homeless population in the parking lot of [organization name], they get quite upset that there’s homeless people hanging around near their property, and they want to call the police. And they just want to not see the substance use problem and homeless problem in the city.”*

#### Lack of medication options

Several informants expressed an inability to offer adequate pharmaceutical alternatives due to the lack of availability of higher-potency prescribed alternatives:

*“There’s so much more we can do, you know, people don’t like hydromorphone, so we’re trying Sufentanil. But what about fentanyl? If that’s what is in the drug supply, doesn’t that make sense that that would be what we would be giving people? Or we’d be able to say to someone, ‘you can have this or that or what works for you’. It’s all a barrier, you know.”*

Some key informants also reported a lack of options for prescribed alternatives that would better match the unregulated opioids that some clients were used to consuming:

*“Because the options that we have available, they’re not perfect substitutions. And particularly around the euphorigenic aspects. And so people are still using street supply.”*

Some key informants reported that a number of clients preferred smoking opioids over injecting or snorting and expressed a need for additional opioid formulations to support different modes of consumption among clients:

*“We have some folks on our waitlist who have declined to join the program because the modality is not their preference. They don’t want to inject or snort. So, there’s a strong preference for smoking. And we’re trying to figure out how we meet this need. But it’s complex trying to develop inhalation spaces—particularly indoor inhalation spaces.”*

To meet clients’ safer supply medication needs, key informants reported relying on their flexible model to increase doses to match different levels of tolerance among clients. Regarding clients’ preferred mode of consumption, there are no smokeable options currently available on the provincial formularies for prescription within safer supply programs.

#### COVID-19-related challenges

Key informants discussed that while the COVID-19 pandemic increased demand for safer supply due to rising overdose deaths following the pandemic’s onset, it also caused several operational challenges for programs, namely staff burnout and difficulty hiring nurses:

*“The potential burnout with the staff having to do iOAT [injectable OAT], do all of their work, plus do COVID swabs and process them. That was a huge impact. And a reason why we had to scale up a bit in nursing staff.”*

Several key informants described difficulties in hiring and retaining staff nurses during the COVID-19 waves. This was particularly prominent in programs that relied heavily on nurses:

*“We’re quite a nurse-led program so our nurses dispense all of our medications and also do primary care. Within the context of a national nursing shortage and COVID-19, it’s pretty hard to hire and retain enough nurses. We have a larger pool of casual nurses, and it’s impossible for us to get our casual nurses to come in to work because they’re working two or three other jobs and they’re completely exhausted. So, that’s been really hard in being able to keep the programs adequately staffed and running.”*

To mitigate the negative impacts of COVID-19 measures, key informants reported leveraging their program’s flexible design to minimize service interruptions by offering take-home doses and easing clinical requirements to reduce the need for multiple clinic visits for observed doses.

## Discussion

In this study, we interviewed key informants from ten different ISSPP in Canada to understand the barriers and facilitators to implementation experienced by these providers. Our thematic analysis identified eight themes related to implementation facilitators and twelve related to implementation barriers. The ISSPP had notable heterogeneity with respect to the degree of ancillary and wraparound services provided. In addition, there were variations across the ten programs regarding eligibility criteria for enrolling clients and the types and range of medications provided. In-depth interviews further revealed that implementation was dynamic and context-specific, as programs monitored and adapted their clinical practices to best meet their clients’ needs during periods of uncertainty and shifting sources of risks for clients.

Consistent with previous research on safer supply implementation, our study found that multidisciplinary staff teams, coordination of service provision, and partnerships with community organizations to organize provision of complementary health and social services all acted as facilitators of implementation [20, 26, 27, 34]. Other facilitators included ongoing needs assessment, program monitoring and evaluation, and integration of clients’ perspectives and preferences in service design. ‘Community buy-in’—the level of support from local interest-holders — was key, consistent with previous research [20, 26]. Evidence from North America’s first supervised consumption sites (SCS) suggests that interest-holder buy-in is critical to overcoming implementation challenges [35–37]. The National Safer Supply Community of Practice, which is a network of healthcare professionals, policymakers, advocates, and people with lived/living experience, has been engaged in knowledge exchange and capacity building to support implementation of safer supply programs [38, 39]. Sustaining these programs likely requires coalition building and mobilization of public opinion based on scientific evidence, strategies that have driven successful implementation of harm reduction services elsewhere [35, 36].

Key informants described wraparound care provision as a crucial implementation facilitator, consistent with previous qualitative evidence [20, 26, 34]. Our study provides further nuance; while wraparound care improved clients’ access to critical services, it also exacerbated organizational challenges. Some programs offering wraparound care faced capacity issues, limiting client enrollment. This aligns with a 2021 survey in British Columbia, where only 16.5% of eligible clients received safer supply [40]. Furthermore, clients with polysubstance use and complex unmet needs required additional support, putting a strain on staff and physical space capacity—both identified as inner setting implementation barriers, similar to our previous national assessment [10]. These findings underscore the need for better planning and sufficient resources to integrate wraparound care effectively.

The perceived lack of support from the colleges of physicians and physicians’ fear of audits were cited as implementation barriers, which echo previous findings about the role of physician professional regulators [23, 27]. Despite statements from the Ontario and British Columbia colleges acknowledging safer supply’s potential clinical value [41, 42], our findings suggest a gap between their professed and actual support. Programs struggled to balance harm reduction approaches with addiction medicine practices like witnessed dosing and urine drug screening. Greater interest-holder engagement is needed to develop consensus-based safer supply guidelines, addressing concerns such as medication diversion and public safety [22, 23]. Context-specific, evidence-based practice guidance is crucial to optimize client benefits and public safety, given the limitations of existing regional directives [6, 43–48].

The need for a prescribed safer opioid supply guideline was underscored by British Columbia’s review of and Health Canada’s expert panel on safer supply [49]. British Columbia’s provincial health officer reported that clinicians call for clear, evidence-informed, client-centred, and flexible guidance. Furthermore, clinicians have highlighted the need for direction on dosage levels and tapering approaches [49]. Guidelines outlining strategies for monitoring client engagement and reporting outcomes relevant to clients, policymakers, and provincial regulators would strengthen safer supply programs in terms of both acceptability to different interest-holders and meeting client needs [50, 51]. Prescription monitoring and risk mitigation strategies are crucial to prevent unintended harms of ISSPP, such as diversion, safer supply-induced toxicity and overdose, and potential reductions in addiction treatment initiation [22]. Nonetheless, balancing standardized guidelines with program flexibility and client-enteredness to avoid a one-size-fits-all approach will remain a challenge that needs to be addressed. Key to addressing this is ensuring appropriate medication type, dosage, and formulary coverage. Studies show that meeting client preferences for dosage and drug type facilitates program engagement [10, 15, 52–54]. Without a sufficient range of prescribed medications, ISSPP may fail to meaningfully reduce overdose risk among those dependent on potent unregulated opioids.

### Strengths and limitations

We used the CFIR framework to provide a comprehensive view of implementation barriers and facilitators across sites and implementation periods. However, our study has limitations. To protect anonymity, we could not collect demographic details or reveal geographic information about key informants. While data came from urban and rural settings, most informants were from medium to large urban communities, limiting the transferability of our findings to other contexts. Furthermore, our results on ISSPP do not apply to other safer supply models, such as risk mitigation guidance [55]. Lastly, as programs were continuously adapting, our findings represent a snapshot of implementation experiences. Future research should explore the long-term sustainability of ISSPP, including how programs evolve over time in response to policy shifts and funding changes. Additionally, studies should examine the perspectives of a broader range of service providers, including rural providers and policymakers, to capture diverse implementation experiences. Comparative studies across different safer supply settings and models are also needed to identify best practices and implementation challenges unique to specific program structures and contexts.

## Conclusions

ISSPP have faced a number of facilitators and barriers to implementation, with the most significant challenges stemming from external factors, including lack of policy support, community pushback, and a complicated regulatory environment. Current and future iterations of safer supply programs need to rely on evidence-based approaches for designing and implementing programs by drawing from existing implementation successes and experiences in the harm reduction field. Moreover, to balance tensions between harm reduction principles and addiction medicine practice, there is a need for evidence-based guidelines, including strategies to mitigate unintended harms in addition to better monitoring and tracking of program outcomes that are relevant to clients, providers, policymakers and provincial regulators. The development of a national practice guideline document driven by consensus between these interest-holders can strengthen future safer supply programs.

## Supplementary Information

Additional file1 (DOCX 57 KB)

## Data Availability

The datasets generated and/or analyzed during the current study are not publicly available to protect the anonymity of study participants.

## Abbreviations

OUD: Opioid use disorder
OAT: Opioid agonist therapy
CDSA: Controlled Drugs and Substances Act
iOAT: Injectable opioid agonist therapy
CFIR: Consolidated Framework for Implementation Research
SUAP: Substance Use and Addictions Program
ISSPP: Integrated safer supply pilot programs
COREQ: Consolidated criteria for reporting qualitative research
SCS: Safe consumption site
