# Supplementary material for: Barriers and facilitators to safer supply pilot program implementation in Canada: a qualitative assessment of service provider perspectives
Source: Harm Reduct J. 2025 Apr 28;22:68. doi: 10.1186/s12954-025-01177-0 (PMC12039088; doi:10.1186/s12954-025-01177-0)
Supplement: Supplementary file 1 — Additional file1 (DOCX 57 KB) [file 12954_2025_1177_MOESM1_ESM.docx]

Barriers and Facilitators to Safer Supply Pilot Program Implementation in Canada: A Qualitative Assessment of Service Provider Perspectives

Consolidated criteria for reporting qualitative studies (COREQ): 32-item checklist

Please indicate in which section each item has been reported in your manuscript. If you do not feel an item applies to your manuscript, please enter N/A.

For further information about the COREQ guidelines, please see Tong *et al.*, 2017: <https://doi.org/10.1093/intqhc/mzm042>

| **No.** | **Item** | **Description** | **Section #** |
| --- | --- | --- | --- |
| **Domain 1: Research team and reflexivity** | | | |
| Personal characteristics | | | |
| *1.* | Interviewer/facilitator | Which author/s conducted the interview or  focus group? | DW & MK |
| *2.* | Credentials | What were the researcher's credentials? *E.g.*  *PhD, MD* | Our multi-disciplinary team consisted of mixed methods researchers, epidemiologists, policy experts, service providers, qualitative researchers, and people with lived/living experience. Credentials of the core research team:  DW & MK hold a PhD.  FN & LM hold a master’s degree. |
| *3.* | Occupation | What was their occupation at the time of the  study? | Co-Principal Investigator DW is a Research Scientist at St. Michael’s Hospital (Toronto, Canada) and holds a dual appointment as Assistant Professor in the Division of Infectious Diseases & Global Public Health at the University of California San Diego and in the Institute of Health Policy, Management & Evaluation at the University of Toronto.  Co-Principal Investigator MK is a Research Scientist at St. Michael’s Hospital and an Assistant Professor at the Dalla Lana School of Public Health, University of Toronto. FN and LM are PhD students and trainees at St. Michael’s Hospital. |
| *4.* | Gender | Was the researcher male or female? | The Core research team members DW, MK, and LM are male. FN is female. |
| *5.* | Experience and  training | What experience or training did the researcher  have? | DW is a drug policy research expert with training in epidemiology. MK is an infectious disease epidemiologist researcher with training in mixed methods research, population health, and implementation sciences.  FN & LM are trained in mixed methods research. |
| Relationship with participants | | | |
| *6.* | Relationship  established | Was a relationship established prior to study  commencement? | No |
| *7.* | Participant knowledge of the interviewer | What did the participants know about the researcher? *E.g. Personal goals, reasons for*  *doing the research* | Participants were aware that the Co-Principal Investigators are substance use and drug policy researchers and appointed scientists at St. Michael’s Hospital, Toronto, Canada. |
| *8.* | Interviewer characteristics | What characteristics were reported about the interviewer/facilitator? *E.g. Bias, assumptions,*  *reasons and interests in the research topic.* | Interviewers introduced themselves (including their professional background and current roles) to the key informants prior to interview commencement. Reason for the research study was discussed. |
| **Domain 2: Study design** | | | |
| Theoretical framework | | | |
| *9.* | Methodological orientation and theory | What methodological orientation was stated to underpin the study? *E.g. grounded theory, discourse analysis, ethnography,*  *phenomenology, content analysis* | We undertook a descriptive qualitative research design and thematic analysis. |
| Participant selection | | | |
| *10.* | Sampling | How were participants selected? *E.g. purposive,*  *convenience, consecutive, snowball* | Purposive sampling was used to recruit study participants. |
| *11.* | Method of approach | How were participants approached? *E.g. face-*  *to-face, telephone, mail, email* | Potential participants were contacted via email. More detail is provided in the methods section in the full paper. |
| *12.* | Sample size | How many participants were in the study? | Our sample size was 10 key informants. More information is provided in the methods section in the full paper. |
| *13.* | Non-participation | How many people refused to participate or  dropped out? What were the reasons for this? | None of the participants refused to participate and none dropped out. |
| Setting | | | |
| *14.* | Setting of data  collection | Where was the data collected? *E.g. home, clinic,*  *workplace* | Zoom interviews were conducted at the home office of Co-Principal Investigators (DW & MK) as outlined in the methods section in the full paper. |
| *15.* | Presence of non-  participants | Was anyone else present besides the  participants and researchers? | There was no one else present besides the participant and interviewer. |

| *16.* | Description of sample | What are the important characteristics of the  sample? *E.g. demographic data, date* | Demographic data is not provided to avoid identifying participants. We have provided participants’ roles in the results section in the full paper. |
| --- | --- | --- | --- |
| Data collection | | | |
| *17.* | Interview guide | Were questions, prompts, guides provided by  the authors? Was it pilot tested? | Yes, each participant was provided the interview guide before the interview date. Interview guide was pilot tested. |
| *18.* | Repeat interviews | Were repeat interviews carried out? If yes, how  many? | No repeat interviews were carried out as each interview was rich. |
| *19.* | Audio/visual recording | Did the research use audio or visual recording  to collect the data? | The interviewers used audio recording to collect data as outlined in the methods section of the full paper. |
| *20.* | Field notes | Were field notes made during and/or after the  interview or focus group? | Since the interviews were audio recorded, the interviewers did not deem field notes necessary. |
| *21.* | Duration | What was the duration of the interviews or  focus group? | Average duration was 75 minutes as noted in the methods section of the full paper. |
| *22.* | Data saturation | Was data saturation discussed? | Data saturation is not applicable. We were funded by Health Canada’s Substance Use and Addictions Program (SUAP) to evaluate eleven programs; of these, we included ten programs in our study. More detail is provided in the methods section of the full paper. |
| *23.* | Transcripts returned | Were transcripts returned to participants for  comment and/or correction? | No, transcripts were not returned to participants for comments. |
| **Domain 3: analysis and findings** | | | |
| Data analysis | | | |
| *24.* | Number of data  coders | How many data coders coded the data? | Two (FN & LM). |
| *25.* | Description of the  coding tree | Did authors provide a description of the coding  tree? | Coding and analysis process is described in the methods section of the full paper. |
| *26.* | Derivation of themes | Were themes identified in advance or derived  from the data? | Themes were derived from the data through inductive coding. Codes were then reviewed to develop themes. Initial themes were reviewed, defined, and named using a deductive process, guided by the consolidated framework for implementation research. More detail is provided in the methods section of the full paper. |
| *27.* | Software | What software, if applicable, was used to  manage the data? | NVivo version 14. |
| *28.* | Participant checking | Did participants provide feedback on the  findings? | Yes, findings were shared with interview participants for member checking and validity as outlined in the methods section of the full paper. |
| Reporting | | | |
| *29.* | Quotations presented | Were participant quotations presented to illustrate the themes / findings? Was each  quotation identified? *E.g. Participant number* | Yes, anonymized quotes are provided throughout the results section alongside author interpretations. |
| *30.* | Data and findings  consistent | Was there consistency between the data  presented and the findings? | Yes |
| *31.* | Clarity of major  themes | Were major themes clearly presented in the  findings? | Yes, major themes were clearly presented. We also presented salient points in our results section. |
| *32.* | Clarity of minor  themes | Is there a description of diverse cases or  discussion of minor themes? | Both common and nuanced responses were considered in developing codes and themes. Our results highlight both major and minor themes, discussing both salient and nuanced narratives shared by study participants. |

Developed from: Allison Tong, Peter Sainsbury, Jonathan Craig, Consolidated criteria for reporting qualitative research (COREQ): a 32-item checklist for interviews and focus groups, International Journal for Quality in Health Care, Volume 19, Issue 6, December 2007, Pages 349–357, <https://doi.org/10.1093/intqhc/mzm042>
